# Selective mycobacterial capture with ultraviolet-polymerized poly-dimethyldiallyl chloride functionalized surfaces

**DOI:** 10.1007/s10856-024-06829-4

**Published:** 2024-09-30

**Authors:** Xuesong Jiang, Bonolo S. P. Mathekga, Digvijay Singh, Devin Coon, Anjana Sinha, Derek Armstrong, Soumyadipta Acharya, Hai-Quan Mao, Yukari C. Manabe

**Affiliations:** 1 Department of Materials Science and Engineering, Whiting School of Engineering, Johns Hopkins University, Baltimore, 21218 MD USA; 2 Institute for NanoBioTechnology, Johns Hopkins University, Baltimore, 21218 MD USA; 3 Translational Tissue Engineering Center, School of Medicine, Johns Hopkins University, Baltimore, 21287 MD USA; 4 Center for Bioengineering Innovation and Design, Johns Hopkins University, Baltimore, 21218 MD USA; 5 Departments of Plastic and Reconstructive Surgery, School of Medicine, Johns Hopkins University, Baltimore, 21205 MD USA; 6 Department of Biomedical Engineering, Johns Hopkins University, Baltimore, 21218 MD USA; 7 Division of Infectious Diseases, Department of Medicine, Johns Hopkins University School of Medicine, Baltimore, 21205 MD USA; 8 Infectious Diseases Institute, Makerere College of Health Sciences, Kampala, Uganda

## Abstract

Tuberculosis (TB) is the top cause of death from a single infectious pathogen after COVID-19. Despite molecular diagnostic advances, two-thirds of the 10 million annual TB cases are still diagnosed using direct smear microscopy which has ~50% sensitivity. To increase the analytical performance of smear microscopy, we developed and characterized a novel polymer (Polydiallyldimethylammonium chloride [PDADMAC]) engraftment on inexpensive polystyrene (PS) specifically functionalized for mycobacterial capture. Engraftment is achieved via UV photopolymerization of DADMAC monomer on plasma-activated PS. The platform was tested on sputum from presumptive TB cases in Kampala, Uganda (*n* = 50), with an increased overall sensitivity of 81.8% (27/33) vs. fluorescent smear microscopy 57% (19/33) compared to a molecular (Cepheid GeneXpert MTB/RIF) gold standard. Frugal smear diagnostic innovation that is rapid and does not require dedicated instrumentation may offer an important solution to bridge the TB diagnostic gap.

**Graphical Abstract:**

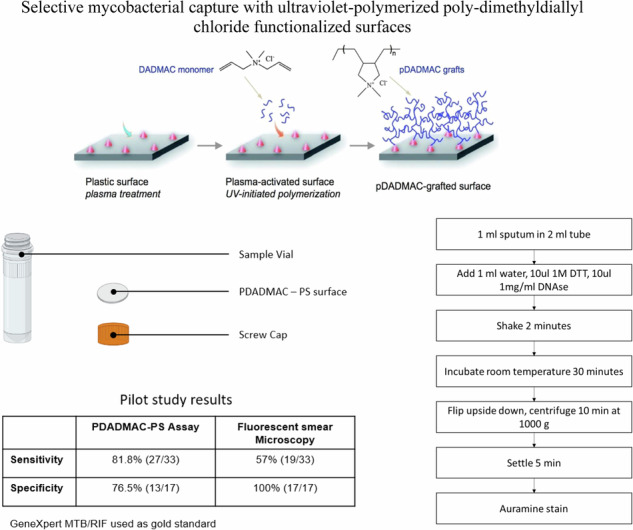

## Introduction

Tuberculosis (TB) is one of the leading causes of infectious disease mortality worldwide. The World Health Organization estimates that the gap between the number of TB cases (~10.6 million) and those diagnosed (7.5 million) is 3.1 million [1]. There were 1.3 million deaths due to TB in 2022, with notable regional differences after the COVID-19 pandemic [1]. Only 59% of recognized cases are microbiologically confirmed, with the vast majority by direct smear microscopy; ~30% are never diagnosed [2]. Smear-negative TB patients can continue to transmit TB to others in the community and have poor treatment outcomes including death, particularly in areas with high HIV prevalence such as sub-Saharan Africa [3]. Smear microscopy was first introduced over 140 years ago and remains the most common and available test in low- and middle-income countries, where 98% of tuberculosis cases occur [1, 4]. In the current pathway for direct smear microscopy, a lab technician smears the patient’s sputum sample onto a slide, heat fixes and stains it, before observing the stained mycobacteria under a microscope. Although it is inexpensive (~$0.20/test) and relatively specific, smear microscopy detects only about 50% of active cases of TB; in children and in people living with HIV (PLHIV), the sensitivity is even lower [4]. A positive direct smear requires at least 10,000 mycobacteria per milliliter of sputum sample [5].

The low sensitivity of this technique can be attributed to the heterogeneity of the sputum sample, and the small proportion of the entire sample that can be smeared onto a slide and examined. Attempts to develop new methods to improve sample quality to achieve a higher concentration of bacteria, such as sedimentation, have not achieved sufficient performance to supplant existing approaches [5–7]. Therefore, a simple, bacterial concentration capture within the sputum sample could improve the sensitivity of smear microscopy.

Mycobacteria are a relatively unusual species of bacteria which are slow growing and difficult to isolate, with the capacity for long periods of dormancy. Most unique, however, is their complex cell wall, composed of long-chain mycolic acids, highly branched, arabinogalactan polysaccharides and cross-linked peptidoglycans, surrounded by a hydrophobic outer membrane of waxes and glycolipids [8, 9]. This cell wall, which is responsible for its resistance to the penetration of most drugs as well as the Gram stain characteristics, has been extensively studied as a target for diagnosis and antibiotic development [10].

The strongly hydrophobic nature of *Mycobacterium spp*. represents a potential target for selective capture as compared to standard Gram-positive and negative bacterial organisms [8, 9]. Bacterial adhesion has been shown to be controlled by both hydrophobicity and the negative electrical potential of the cell surface, with electrostatic effects being of minor significance in strongly hydrophobic bacteria [11, 12].

We sought to create and optimize an inexpensive, robust, polymer film that could maintain optical transparency while selectively binding to *Mycobacterium spp*. to concentrate bacteria, and thereby increase diagnostic sensitivity. Such a film could be used in independent assays or for sample enrichment for downstream diagnostics such as smear microscopy, culture, or molecular tests. Here, we describe the preparation and characterization of a poly (diallyldimethylammonium chloride)-grafted polystyrene (PDADMAC-PS) film that shows significant affinity for mycobacteria. To assess performance in real-world scenarios, a device with the polymer-treated surface was used to enrich samples prior to smear microscopy examination. Discarded sputum samples from patients suspected to have TB in Kampala, Uganda were pilot-tested for proof-of-concept for sample enrichment.

## Materials and methods

### Reagents and test organisms

All chemicals and reagents were purchased from Sigma-Aldrich (St Louis, MO, USA) unless otherwise specified. IKEPLUS-RFP, a strain of *Mycobacterium smegmatis* transfected with *Mycobacterium tuberculosis* genes and expressing red fluorescent protein (RFP) commonly used as a non-pathogenic mycobacterial *M. tuberculosis* substitute, was used as the primary test organism [13]. It was kindly provided by Professor John Chan at Department of Microbiology and Immunology, Albert Einstein College of Medicine, Bronx, New York, USA. In brief, IKEPLUS were grown on a nutrient agar at 36 ^∘^C for 16 h to generate isolated colonies. A loopful of cells freshly grown on 7H10 agar plates were inoculated into 5 mL of trypticase soy broth (Mediatech, Manassas, VA, USA) and was shaken for 16 h at 36 ^∘^C and 230 rpm. *M. smegmatis* cultures for infection were grown from low-passage freezer stocks to mid-log phase, sub-cultured and grown to an A600 of 0.1–0.5, washed, and re-suspended in PBS plus 0.05% Tween-80 (PBS/T). Bacteria were sonicated before experiments to obtain single-cell suspensions.

### Polymer surface treatment and PDADMAC grafting

Black polystyrene (PS) 96-well plates (Corning, NY, USA) were oxygen plasma-treated at low pressure (200–600 mtorr) for 10 min. Poly (dimethyl diallyl chloride) (PDADMAC) was grafted onto the PS surface by photo-activated surface-grafting free radical polymerization. DADMAC monomer (100 μL) was dispensed into each well. The temperature of the solution was maintained at 20 ^∘^C by cooling the dish in an ice bath. Samples were then exposed to ultraviolet (UV) light from a 400-W mercury lamp manufactured by Dymax (Torrington, CT, USA), with a wavelength of ~320–390 nm. The intensity and the energy of UV light were measured using the Accu-Cal 50 radiometer from Dymax (Torrington, CT, USA). Light intensity ranging between 22-24 *mW*/*cm*^2^ was used. The PDADMAC-grafted PS surfaces were then thoroughly washed with deionized water to remove any un-grafted PDADMAC from the surface of the plate.

### Surface characterization of charge density using a colorimetric assay

The acid orange-7 staining colorimetric method was used to quantify the amount of exposed PDADMAC on the PS surface. A 0.3-mL dye solution (14 mg/mL) in acidic solution (Milli-Q water adjusted to pH 3 with 1 M HCl) was dispensed into each well of the PS plate and shaken for 60 min at 100 rpm. The samples were then intensively rinsed 3 times using the acidic solution (pH 3) to remove unbound dye. Once air-dried, aliquots of 0.1 mL alkaline solution (0.25 M sodium carbonate, pH 11.25) were added into each well and mixed on a shaker for 10 min. The absorbance of the solution was then measured on a BioTeks Synergy 2 Microplate Reader at 484 nm post standardization.

### Surface adhesion test using red fluorescent protein (RFP)-expressing IKEPLUS cells

A suspension of 1.0 × 10^6^ IKEPLUS-RFP cells were washed in 50 mL of PBS twice. Cell concentrations were determined by hemocytometer under the Axiovert 40CFL, Carl Zeiss inverted fluorescent microscope (Jena, Germany) and diluted to the desired concentration. A 50 μL, 100 μL, or 200 μL of cell suspensions were added into the PDADMAC-PS plates and centrifuged at 1000 *×* *g* for 10 min. Unbound cells were removed by washing and shaking at 90 rpm for 1 min (New Brunswick, Edison, NJ, USA). Cell images were captured using a microscope to examine the number of cells attached to the modified surfaces. Unless otherwise specified, 100 μL water was added to each well and the fluorescent intensity was measured using a BioTeks Synergy 2 Microplate Reader at Ex/Em = 540 nm/590 nm.

### Cell count conversion

Preparation of standard cell concentrations was done using a standard calibration curve. Triplicates of 1.0 × 10^6^ IKEPLUS-RFP cells/mL solutions were created. These suspensions were plated in 100 μL volumes in a 96 well plate, along with serial dilutions of 1:25, 1:50, 1:100, 1:200, 1:400, 1:800, and 1:1600. A standard curve was generated using fluorescent intensities against concentration.

### Statistical analysis

Each data point represents the average of the readings from two to three independent experiments. Significant differences were determined by the Student’s *t* test. A *p* value of <0.05 was considered as significant.

### Proof-of-concept study workflow

A different workflow was used in the proof-of-concept study, compared to that followed in lab settings. To accommodate the equipment available at the study site, we grafted the polymer film onto a PS-septum. Moreover, the use of clinical samples required an additional liquefaction and decontamination step (achieved using dithiothreitol and DNAse). The PS-septum was placed inside a screw cap (Fig. 4A).

In total, 1 mL water, 10 μL 1M dithiothreitol (DTT), 10 μL 1 mg/mL DNAse were added to 1 mL of sputum in a 2 mL tube. The solution was shaken for 2 min and incubated for 30 min (Fig. 4B). The screw cap of the vial contained a removable septum with the functional PDADMAC-PS surface on the underside. The vial was inverted and centrifuged for 10 min at 1000 × *g*. After settling for 5 min, the septum was removed, stained using Auramine O staining technique (as per standard procedure [14]), and viewed under a fluorescent microscope.

## Results

### Plastic slide material selection and engraftment of PDADMAC

**Fig. 1:**
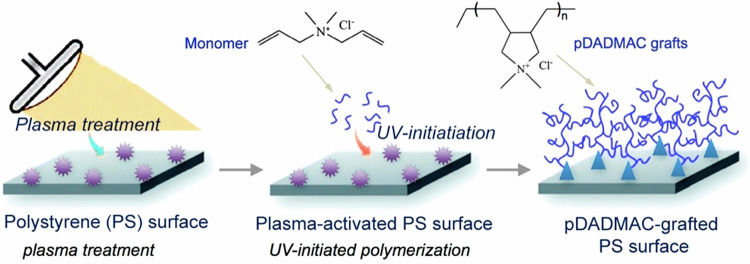
Schematic of PDADMAC surface conjugation onto PS surface, illustrating plasma pre-activation (purple stars) followed by UV-initiated polymerization (blue triangles)

PDADMAC has previously been shown to have high affinity for mycobacteria [15–17]. Monomer and polymer dip-coated glass results in mycobacterial clumping (Fig. 5a), therefore, we selected two inexpensive plastic materials, polyethylene terephthalate (PET) and PS for testing; PET had an unacceptably high fluorescence background. To immobilize polymer on PS, low-temperature air plasma treatment for 90 s generated a reactive alkyl surface (Fig. 1). UV cross-linking engraftment was used to increase the grafting efficiency of the PDADMAC which showed optimal bacterial distribution when using 20% weight/volume DADMAC on PS (Fig. 5b).

### Optimization of PDADMAC surface for mycobacterial capture

**Fig. 2:**
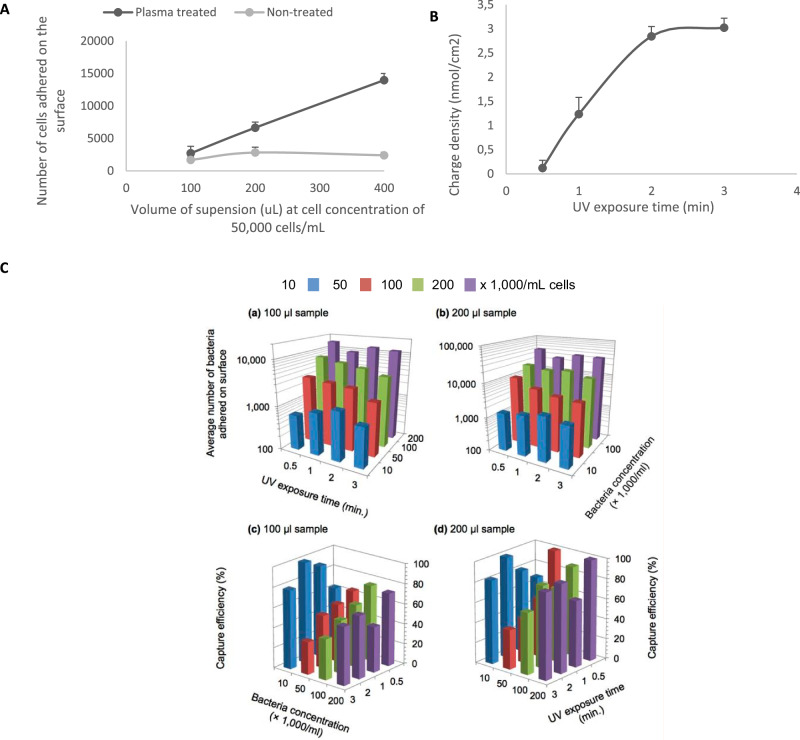
**A** The effect of low-temperature plasma treatment prior to polymerization on mycobacterial cell adhesion at varying volumes (uL) of cell suspensions put in each well. The cell concentration is 50,000 cells/mL. **B** Quantification of polystyrene surface charge density after polymer coating via binding of acid orange 7 to PDADMAC. **C** Capture efficiency for PDADMAC-grafted slides as a function of UV exposure time (i.e., amount of polymerization), and bacterial concentration. 100 uL and 200 uL sample volume were used. The current detection limit for direct smear microscopy tests is at 10,000 bacilli/mL concentration

Using fluorescently tagged IKEPLUS-RFP *Mycobacterium smegmatis* to assess the capture efficiency, plasma-treated surfaces improved mycobacterial adherence (Fig. 2A). UV treatment time exposure times of 0.5, 1, 2, and 3 min were evaluated (Fig. 2B). Adherence testing was performed across samples with varying mycobacterial concentrations (10,000–200,000 CFU/mL) and volumes (50–200 μL) applied (Fig. 2C). UV exposure time of 2 min optimized the positive charge density on the surface for mycobacterial capture across various mycobacterial cell concentrations. Saturation was reached after 3 min. All surfaces were capable of successful capture, with higher concentrations leading to higher capture rates as expected, without an obvious correlation between polymer density and capture performance present at high concentrations of bacteria (Fig. 2C). At the smear microscopy limit of detection of 10,000 CFU/mL, the 2-min UV exposure time had the highest number of adhered bacteria.

### Centrifugal force is required for mycobacterial capture onto the engrafted surface

Microscopic observation suggested that it is important to maximize the direct contact of the mycobacteria with the surface to ensure sufficient capture. The mycobacteria had a strong tendency towards self-aggregation and clustering [18], but after dispersion onto the polymer they remained immobilized. The polymer-bacteria interaction consistently led to uniformly distributed bacteria across the slide, compared with the glass slide, which had areas of bacterial clumping (Fig. 5a). Note that this does not necessarily indicate the homogeneity of polymer grafted.

**Fig. 3:**
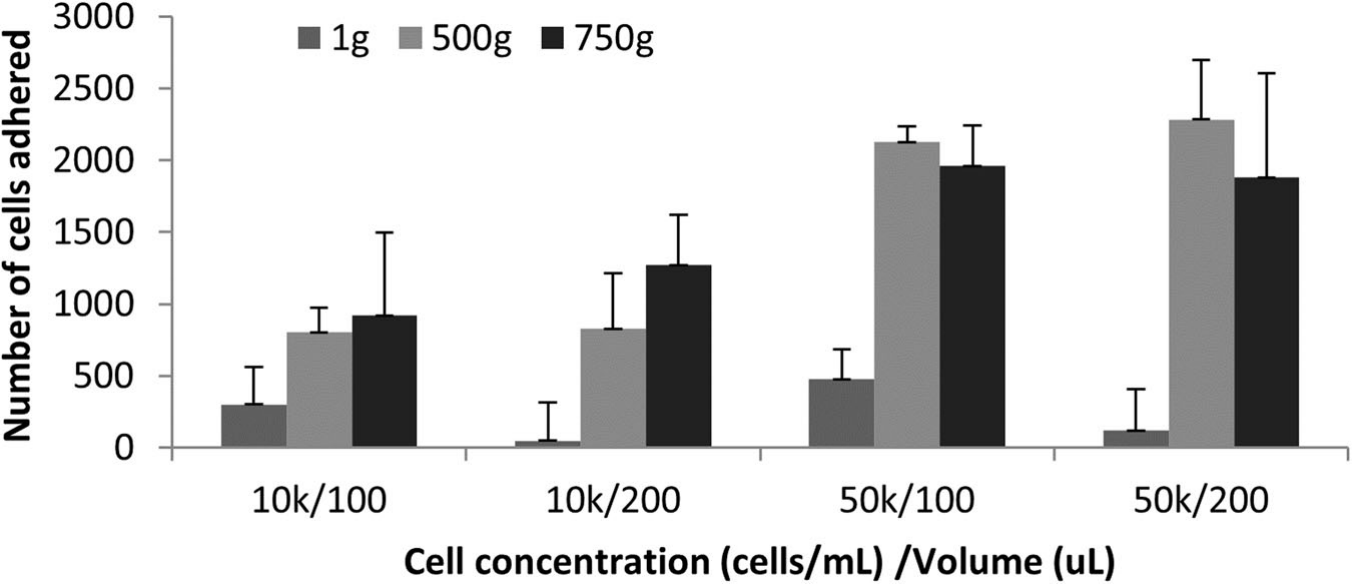
The effect of 10 min of centrifugation (at 1 × *g*, 500 × *g*, and 750 × *g*) on IKEPLUS-RFP mycobacterial cell adhesion over different cell concentrations (cells/mL) and volumes (uL). In the first 2 sets of 3 bars, 100 μL and 200 μL of 10,000 mycobacteria/mL solution was added, respectively. The second two sets of bars had a higher concentration of bacteria (50,000 cells/mL)

**Fig. 4:**
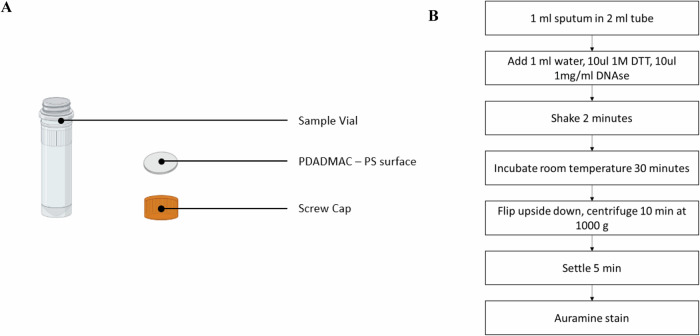
**A** Exploded view of screw cap with removable PDADMAC-PS septum. Created with BioRender.com (**B**) Flowchart depicts proof-of-concept study protocol

**Fig. 5:**
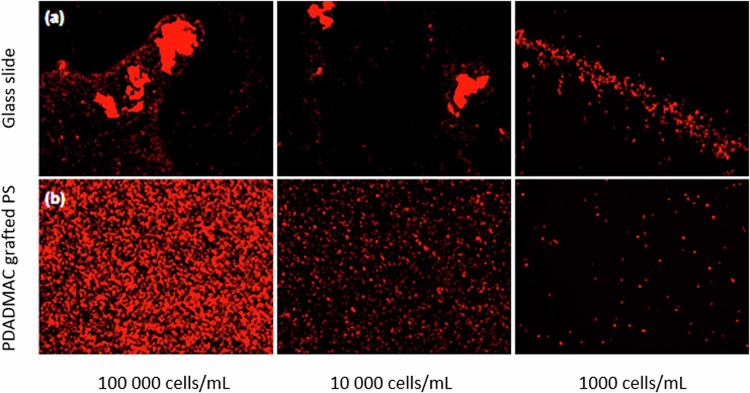
Fluorescent images of IKEPLUS-RFP M. smegmatis cells adhered on (**a**) glass slides or (**b**) PDADMAC grafted polystyrene across varying mycobacterial cell concentrations. We compared it to glass slide microscopy prepared by the standard method (i.e., drop dried on the slide). The polymer-bacteria interaction consistently led to uniformly distributed bacteria across the slide, whereas the glass slide showed clumping

We then varied centrifugation speeds using various concentrations of bacteria in different volumes. Centrifugal force as low as 500 × *g* is required to drive the mycobacteria into the positively charged polymer surface for binding to occur (Fig. 3). A more modest increase in capture occurs with increasing centrifugation speeds. After dispersion onto the polymer, the mycobacteria remained immobilized. The polymer-bacteria interaction consistently led to uniformly distributed bacteria across the slide (Fig. 5a). Based on this data and the speeds achievable with hand-crank and small battery-operated centrifuges, we selected 1000 × *g* for use in the proof-of-concept study.

### Selective mycobacterial capture in the presence of E.coli and in mucin matrix

**Fig. 6:**
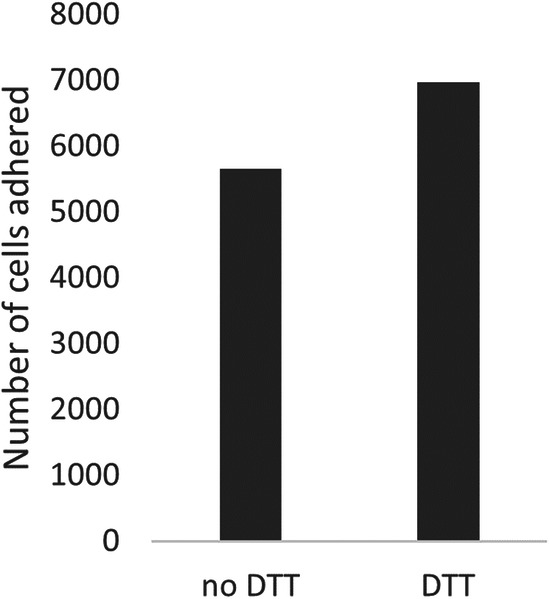
Sputum is a challenging medium for bacterial binding, with significant quantities of charged macromolecules present in solution. We evaluated the impact of suspending the bacteria in a 2% mucin sample representative of sputum rather than water. Mucolytics are commonly used for the preparation of clinical sputum samples, so we also evaluated the addition of a 1:1 ratio of 2% dithiothreitol (DTT) and MES buffer as a mucolytic agent. Bacterial capture increased with the use of DTT

**Fig. 7:**
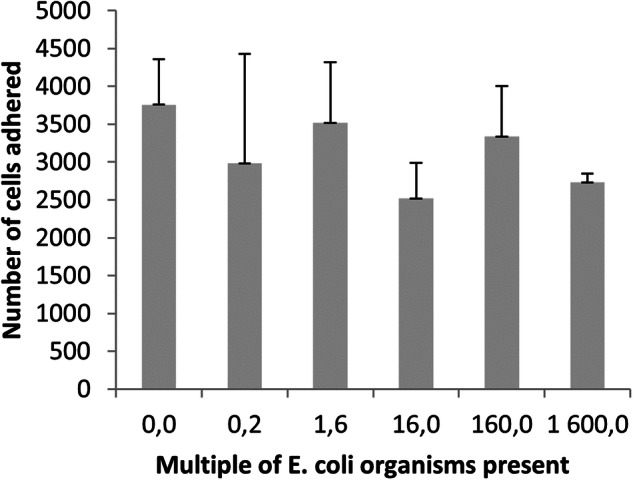
Graph of bacterial yield in the presence of varying quantities of E. coli contaminant organisms. The concentration of mycobacterium used was 50K CFU/mL. The PDADMAC-polystyrene interface continues to capture IKEPLUS-RFP mycobacteria even in the presence of a 1600-fold excess of *E. coli*

To test the polymer-engrafted surface in an artificial mucin matrix to simulate the viscosity of sputum, a series of tests were conducted using mucin samples spiked with different concentrations of mycobacteria. The surface was still able to capture mycobacteria in a mucin matrix, although the addition of 2% DTT and MES buffer as a mucolytic agent led to slightly improved bacterial capture (Fig. 6). Next, to test the selectivity of mycobacterial capture, increasing concentrations of E. coli were added to the mucin matrix with a consistent concentration of mycobacteria with little appreciable loss in mycobacterial capture (Fig. 7).

### Proof-of-concept testing

To assess performance using clinical samples, the prototype surface was assessed in a proof-of-concept study at the Infectious Diseases Institute at Makerere University College of Health Sciences in Kampala, Uganda. Please note that the aim was to evaluate the polymer’s efficacy in capturing MTB using clinical samples. We did not seek to investigate clinical workflow benefits (i.e. cost and time savings, workflow optimization, etc.). Fifty de-identified sputum sample discards from presumptive TB patients whose samples had been tested using traditional sputum fluorescence smear microscopy (i.e., Auramine O staining without the polymer assay) and Cepheid’s GeneXpert MTB/RIF (Sunnyvale, CA, USA) test were frozen at –80 ^∘^C. GeneXpert testing was performed as per the manufacturer’s instructions. The 50 samples used in the study (smear scanty, negatives, and positives) were selected based on specimens originally submitted to the institute for TB testing. The de-identified sputum specimens were thawed and then tested; the lab technician performing the testing was blinded to the GeneXpert and traditional fluorescent smear microscopy results.

Of 50 samples tested, 33 (66%) and 17 (34%) were GeneXpert MTB/RIF positive and negative, respectively. Sputum smear fluorescence microscopy had a sensitivity of 57.6% (19/33) and specificity of 100% (17/17). In contrast, the PDADMAC-PS system yielded 81.8% sensitivity (27/33) with 76.5% specificity (13/17) due to 4 false positives compared to GeneXpert MTB/RIF; these samples were noted to be mucopurulent.

Of the 33 positive samples based on GeneXpert assay, 14 were negative by smear microscopy. The PDADMAC-PS assay successfully detected MTB in 10 of these 14 cases, as shown in Table 1. Results of the PDADMAC-PS system were graded (negative, scanty, 1+, 2+, 3+) based on the International Union Against Tuberculosis and Lung Disease (IUATLD)/WHO guidelines for reporting microscopy results [19]. The PDADMAC-PS assay was able to detect positive samples starting from GeneXpert low positives

**Table 1:** GeneXpert-positive, smear microscopy-negative samples that were successfully detected (positive) by the PDADMAC-PS system

GeneXpert assay		PDADMAC-PS assay	
Grading scale	Number of positive samples	Grading Scale	Number of positive samples
Very low/Trace	2	N/A	0
Low	4	Scanty	3
Medium	6	1+	6
High	2	2+	1
Total samples	14	Total samples	10

## Discussion

PDADMAC is a high-charge density, cationic polymer used for a variety of applications including wastewater processing [20]. It is useful as a flocculant and promotes aggregation and clustering [21]; it has been used to capture mycobacteria in a magnetic bead format [15, 17]. PS is a commonly used aromatic polymer that offers excellent mechanical performance and optical clarity and is inexpensive. We found that plasma pretreatment to activate the surface of PS was necessary to achieve effective PDADMAC binding. Oxygen species in the plasma likely chemically interact with the polystyrene surface to create polar groups like hydroxyl, carbonyl, epoxy, peroxide, ester, and carboxyl, resulting in a changed surface wettability, i.e. decreased water contact angle. The water contact angle (wettability) of the plasma-treated surface may quickly change over time, depending on the treatment and storage conditions. Hence, photoinitiated free radical polymerization was employed to graft PDADMAC onto the polystyrene surface, a process crucial for determining the modified surface’s performance. Optimization of the conditions for this step was based on enhancing bacteria capture efficiency. When followed by UV irradiation to generate reactive radicals, the resulting process yielded polymerization and extension of long PDADMAC graft chains onto the aliphatic PS.

We observed that some degree of perpendicular force was necessary for significant capture, though binding appeared to be a largely irreversible phenomenon; the bacteria remained adherent even after repeated washing. This can be explained by the tendency of mycobacteria to cluster together in the absence of external force [22]. Less immediately obvious is the explanation for the highly effective binding between PDADMAC and mycobacteria, given the high cationic charge density of this polymer. The mycobacteria also show little affinity for a very hydrophobic, unfunctionalized PS surface [23]. Even the presence of a more negatively charged bacterial competitor such as E. coli did not significantly affect the binding efficiency.

One potential hypothesis is that the test organism, IKEPLUS-RFP, possesses cell walls comprising mycolic acids, long-chain fatty acids imparting a negative charge to the cell surface. PDADMAC, a cationic polymer with high-charge density, interacts electrostatically with the negatively charged cell surface. The interaction between grafted polymer and cell does not necessitate cell wall penetration. Another larger-scale phenomenon could be a bilayer construct with the mycobacteria preferentially associated with the hydrophobic PS, thermodynamically trapped against the solid surface by highly charged long PDADMAC chains separating the bacteria from free solution. We were able to show that the system worked in the presence of physiologic salt solutions and mucin (Fig. 6). In addition, mycobacterial capture remained optimized in the presence of increasing proportions of E. coli (Fig. 7).

In a limited pilot study on sputum discards from presumptive TB patients, we showed promising data that this sample enrichment device could increase the sensitivity of sputum microscopy with only a few workflow modifications. The system yielded a significantly enhanced sensitivity of 81.8% over 57.6% from the existing fluorescence microscopy workflow. There was also a dose-response increase in capture with increasing sputum smear positivity. An optimized workflow, such as combining steps or batch-wise procedures, while not in the purview of this paper, can significantly improve clinical lab outputs, especially at TB microscopy centers. It is important to note that in most markets today, smear microscopy is still offered at about one-tenth of molecular prices, with insensitive performance. Enrichment-enhanced solutions utilizing surfaces such as this one have the potential to provide high-performance testing at low prices and reduce TB underdiagnoses. Furthermore, enrichment of paucibacillary samples, such as those from children, HIV patients, or even new sample types such as saliva, can alleviate current challenges with performance.

**Fig. 8:**
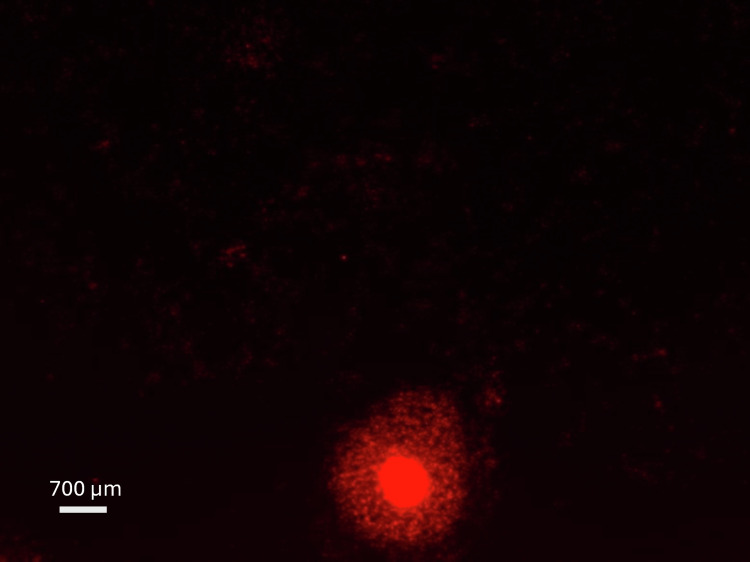
Bacterial capture on PDADMAC-grafted polystyrene, where an aluminum mask with a 700 μm hole was used during UV-irradiation to create an isolated area of PDADMAC. IKEPLUS-RFP mycobacteria preferentially concentrated in the area of the mask after application (cell concentration 50 K/ml)

One important limitation of this pilot proof-of-concept study is that we did not use culture as the gold standard. When compared to a culture gold standard in TB-endemic countries, GeneXpert has imperfect sensitivity in smear-negative TB suspects. Boehme et al. [24] found that GeneXpert MTB/RIF test sensitivity was only 76.9% in smear-negative, culture-positive patients overall (296 of 385 samples); furthermore, in Uganda, the same location as our study, sensitivity was only 57.7% [24]. Therefore, it is possible that the 4 false positives compared to GeneXpert found in our pilot study were indeed true positives. Unfortunately, culture testing is not routinely performed in Uganda unless rifampin resistance is detected by GeneXpert. One potential drawback of this diagnostic device is the need for centrifugation, especially in resource-limited settings where this concentration capture microscopy could be most useful. Additional testing, determining if shorter centrifugation times using small battery-operated centrifuges will be needed. However, the additional time needed for centrifugation is offset by time-savings in microscopy as mycobacteria are focused on a much smaller area (Fig. 8).

Finally, we did not evaluate bacterial viability after capture, which would be very useful in decreasing the time needed to culture mycobacteria and determining phenotypic drug susceptibility in smaller health facilities. Detection of multi-drug-resistant tuberculosis (MDR-TB) is an area of great clinical concern and is time-consuming to diagnose [25]. However, new tests are being developed to conduct molecular resistance testing on smear slide specimens by examining growth after antibiotic exposure, which could potentially represent a valuable extension of our system [26].

## Conclusion

We leverage existing smear microscopy workflow and increase its sensitivity with concentration capture. Polymer-treated inexpensive plastic surfaces can concentrate sputum TB bacilli onto a focused area that can be quickly examined using smear microscopy with significantly higher sensitivity for the detection of Tuberculosis. This may be a more sustainable and affordable approach, particularly in high-burden countries with limited resources. Frugal smear diagnostic innovation that is rapid and does not require dedicated instrumentation may offer an important solution to bridge the TB diagnostic gap.
